# Association of family function and suicide risk in teenagers with a history of self-harm behaviors: mediating role of subjective wellbeing and depression

**DOI:** 10.3389/fpubh.2023.1164999

**Published:** 2023-06-02

**Authors:** Mo Chen, Yang Zhou, Dan Luo, Shu Yan, Min Liu, Meng Wang, Xin Li, Bing Xiang Yang, Yi Li, Lian Zhong Liu

**Affiliations:** ^1^Department of Psychiatry, Wuhan Mental Health Center, Wuhan, Hubei, China; ^2^Department of Psychiatry, Wuhan Hospital for Psychotherapy, Wuhan, Hubei, China; ^3^Center for Wise Information Technology of Mental Health Nursing Research, School of Nursing, Wuhan University, Wuhan, China

**Keywords:** history of self-injury, suicide, family relationship, children and adolescents, chain mediation

## Abstract

**Background:**

A history of self-harm behaviors is closely associated with subsequent suicide death. Although many factors associated with suicide have been identified, it remains unclear how these factors interact to influence suicide risk, especially among teenagers with a history of self-harm behaviors.

**Methods:**

Data were collected from 913 teenagers with a history of self-harm behaviors through a cross-sectional study. The Family Adaptation, Partnership, Growth, Affection, and Resolve index was used to assess teenagers' family function. The Patient Health Questionnaire-9 and the Generalized Anxiety Disorder-7 were used to evaluate depression and anxiety in teenagers and their parents, respectively. The Delighted Terrible Faces Scale was used to assess teenagers' perception of subjective wellbeing. The Suicidal Behaviors Questionnaire-Revised was used to evaluate teenagers' suicide risk. Student's *t*-test, one-way ANOVA, multivariate linear regression, Pearson's correlation, and a structural equation model (SEM) were applied to data analysis.

**Results:**

Overall, 78.6% of teenagers with a history of self-harm behaviors were at risk for possible suicide. Female gender, severity of teenagers' depression, family function, and subjective wellbeing were significantly associated with suicide risk. The results of SEM suggested that there was a significant chain mediation effect of subjective wellbeing and depression between family function and suicide risk.

**Conclusion:**

Family function was closely associated with suicide risk in teenagers with a history of self-harm behaviors, and depression and subjective wellbeing were sequential mediators in the association between family function and suicide risk.

## 1. Introduction

Self-harm is a general term for non-fatal self-injury or self-poisoning with or without suicidal intention (1). Based on the existing suicidal intention or not, self-harm can be classified into suicide attempt and non-suicidal self-injury (NSSI) (2). According to the International Classification of Diseases 10th edition (ICD-10), deliberate self-harm was identified with at least one code X60 to X84 (3). To differentiate self-harm without suicidal intention from self-harm with suicidal intention, the United States developed a clinical modification based on WHO's ICD-10 (ICD-10-CM), and a new diagnosis code R45.88 was added (4). Self-harm is a focus of concern among children and adolescents. The onset of self-harm behaviors frequently occurs between the age of 12 and 14 years, while the prevalence rises during middle adolescence (5). Meta-analyses showed that the global lifetime prevalence of self-harm behaviors and NSSI in children and adolescents were 13.7% (95% CI: 11.0–17.0%) and 22.1% (95% CI: 16.9–28.4%) (6). In China, 2.7% and 8.8% of adolescents attempted suicide and experienced NSSI, respectively (7). Although self-harm behaviors decline substantially over time, these behaviors can develop into chronic practice in some individuals, even extending into late adolescence and adulthood (5). A report in the *Lancet Psychiatry* has estimated that compared to the age cohorts above 20 years, self-harm behaviors among adolescents lead to the largest loss of potential years of life (65.1 years) and productive years of life (48.7 years), as well as the largest cost derived from full incapacity and fatality (8). However, psychological therapeutic trials such as dialectical behavioral therapy have been proposed to be a valuable treatment in reducing children and adolescents' self-harm (9, 10). Therefore, adolescence is a crucial developmental stage for early intervention and the prevention of self-harm behaviors.

It is worth noting that individuals who had previous self-harm behaviors are at considerable risk for future suicide. It has been estimated that ~50% of teenagers who died by suicide had previously self-harmed (11). A prospective cohort study also showed that the 12-month incidence rate of suicide in children and adolescents with previous non-fatal self-harm behaviors was more than 30 times higher than the expected rate in the general population, and the risk might persist over several years (12). Suicide is one of the major causes of death in children and adolescents, and has been identified as a severe public health problem worldwide (13). Epidemiologic studies showed that the prevalence of suicide ideation among adolescents ranged from 19.8 to 24.0% and increased rapidly between the ages of 12 and 17 years, and the lifetime prevalence of suicide attempts ranged from 3.1 to 8.8% and increased during early to mid/late adolescence (14). Even though many psychological tools can help evaluate the risk of suicide, it is still challenging to predict episodes of suicide, especially after non-fatal self-harm behaviors. To date, there are no appropriate tools for predicting a high risk of suicide in adolescents (15). However, suicide is preventable and the adolescence is a critical period for prevention, during which more years of life can be saved by timely intervention (14). Therefore, exploring the causes and patterns of suicide risk in children and adolescents can provide opportunities to intervene on this trajectory earlier in life.

Suicide has been revealed to be the result of interactions among biological, psychological, and environmental factors. Regarding biological aspects, most suicidal patients were reported as having a dysregulated hypothalamus-pituitary-adrenal axis (16). Neurobiological evidence has revealed that the pathogenesis of suicide is related to neuroinflammation in the brain which activates the kynurenine pathway with subsequent serotonin depletion and the stimulation of glutamate neurotransmission, which are accompanied by decreased brain-derived neurotrophic factor levels (17). In suicidal young adults, several brain circuits appeared to be atypical and altered, such as functional connectivity in the cerebral cortex, limbic system, and cerebellum (18). Beyond that, the social environment is another essential factor influencing suicide risk. The suicide of children and adolescents might be prevented by improving social support across the domains of family, school, and friends (19–21). Comparisons between the relative contributions of peer, family, and school support suggested that family support seemed more important in understanding suicide risk (22). Family context and family relationships are identified as risk factors for suicide. It has been proposed that parental psychopathology, poor parental monitoring, and family discord are linked to adolescent suicide (23), while family cohesion, family expressiveness, and perceived responsibilities to the family are identified as potential protective factors against adolescents' suicide (24). These studies implied that the reinforcement of family function could reduce suicide risk in children and adolescents. Approximately 90% of people who had suicide attempts or suicide behaviors had psychiatric disorders (25). Particularly, problems with emotion regulation are differentially connected to suicide, depressive symptoms are proposed to be the most probable risk factors for suicide behaviors, and anxiety symptoms are significantly associated with suicide ideation and suicide attempt (26, 27). Subjective wellbeing refers to an individual's satisfaction with material possessions, health, achievements, relationships, safety, social connectedness, and future security (28), which includes emotional, psychological, social, and spiritual aspects (29). A negative association between subjective wellbeing and suicide rates has been found in several studies (30–32). Analyzing mental health from risk aspects such as depression and anxiety as well as from protective aspects such as wellbeing can improve understanding of the effect of mental health on suicide.

Although many factors associated with suicide have been identified, it remains unclear how these factors interact to influence suicide risk, especially among teenagers with a history of self-harm behaviors. Therefore, this study aims to identify factors associated with suicide risk among teenagers with a history of self-harm behaviors and further investigate the mediating role of emotional disorders and subjective wellbeing between family function and suicide risk. The proposed hypotheses are: (1) family function is negatively associated with suicide risk; (2) subjective wellbeing mediates the association between family function and suicide risk; (3) emotional disorders (i.e., depression and anxiety) mediate the association between family function and suicide risk; and (4) subjective wellbeing and emotional disorders play a chain mediation role between family function and suicide risk.

## 2. Methods

### 2.1. Participants

Our research was part of the Students' Mental Health Network (SMHN), a project carried out in Wuhan, China. A cross-sectional study was performed and participants along with a primary caregiver were recruited from four junior high schools (grades 7–9) and four senior high schools (grades 10–12) by using a cluster sampling method. Inclusion criteria were: (1) between 12 and 18 years of age; and (2) could be individually matched with their primary caregivers. Exclusion criteria were: (1) history of neurological, psychiatric, or other serious somatic diseases; (2) caregivers were not father or mother; (3) unwilling to participate; and (4) nationality was not Chinese. Participation in the study was voluntary. All participants completed informed assent and their parents completed informed consent.

Participants were asked to complete the self-administered anonymous questionnaires using a computer located in the computer room of their school and under the guidance and supervision of teachers. The survey took 10–20 min to complete. Participants were informed that data would remain anonymous and available only to the researchers. All participants were first asked if they had a history of self-harm behaviors: “In the past 12 months, have you had self-harm behaviors without any intention of committing suicide?” (33). A total of 8,990 participants completed the survey and 913 (10.16%) reported a history of self-harm behaviors. This group was then assessed for suicide risk.

The investigation was approved by the Ethics Committee of the Wuhan Mental Health Center (Approval No. KY2021.11.01).

### 2.2. Measures

#### 2.2.1. Suicide risk

The Suicidal Behaviors Questionnaire-Revised (SBQ-R) was applied to screen for suicide risk among teenagers with a history of self-harm behaviors. The SBQ-R contains four items: (1) lifetime suicide intention/attempt; (2) frequency of suicide intention over the past 12 months; (3) telling someone else about suicide intention; and (4) likelihood of attempting suicide someday. The total score of the SBQ-R ranges from 3 to 18 and a higher score indicates a greater risk of suicide. Generally, a cutoff score of 7 was set to identify the possibility of suicide. The SBQ-R has shown good reliability and validity in Chinese college students (34).

#### 2.2.2. Family function

The Family Adaptation, Partnership, Growth, Affection, and Resolve (APGAR) index developed by Smilkstein was applied to evaluate family function by assessing an individual's satisfaction with their family relationship. The scale is a valid and reliable measurement that includes five items scored on a 3-point Likert scale of 0–2. The total score is obtained by summing the scores of five items with a higher total score indicating better family function. The Family APGAR has well-established reliability and validity in Chinese adolescents (35, 36).

#### 2.2.3. Depression

The 9-item Patient Health Questionnaire (PHQ-9) was applied to evaluate for depression. The items relate to symptoms experienced during the past 2 weeks. Each item is scored on a 4-point Likert scale of 0 to 3 with a higher total score indicating more severe depressive symptoms. The PHQ-9 has demonstrated strong reliability and validity in Chinese adolescents (37).

#### 2.2.4. Anxiety

The 7-item Generalized Anxiety Disorder (GAD-7) was applied to evaluate for anxiety. The items relate to symptoms experienced during the past 2 weeks. Each item is scored on a 4-point Likert scale of 0 to 3 with a higher total score indicating more severe generalized anxiety. The GAD-7 has well-established reliability and validity in Chinese adolescents (37).

#### 2.2.5. Subjective wellbeing perception

An individual's subjective perception of wellbeing was measured via the Delighted Terrible Faces Scale (DTS). This is a single-item satisfaction scale that contains seven response faces ranging from smile to frown with the score ranging from 7 to 1 (38). A higher score reflects a higher sense of wellbeing.

### 2.3. Statistical analysis

Data analysis was performed using SPSS version 22.0. Student's *t*-test and one-way ANOVA were used to compare suicide risk. Multivariate linear regression was used to identify factors associated with suicide risk. Pearson's correlation was used to analyze the correlations between psychosocial factors statistically associated with suicide risk.

A structural equation model (SEM) was constructed using Mplus version 8.0 to explore the relationship among family function, subjective wellbeing, emotional disorders, and suicide risk. In SEM, the root mean square error of approximation (RMSEA) value is <0.08, and comparative fit index (CFI) and Tucker Lewis index (TLI) values are >0.90, indicating acceptable models.

All statistical tests were two-sided and *P* < 0.05 was considered statistically significant.

## 3. Results

### 3.1. Suicide risk in teenagers with a history of self-harm behaviors

A total of 913 participants indicated having a history of self-harm behaviors, including 394 (43.2%) junior high school students (grades 7–9) and 519 (56.8%) senior high school students (grades 10–12). When a cutoff score of 7 was set to identify the possibility of suicide, nearly 78.6% (718/913) of students were regarded to possess the possibility of suicide.

In the following analysis, the total score of SBQ-R was applied to indicate suicide risk. As shown in Table 1, among participants with a history of self-harm behaviors, females and teenagers who had not lived with their parents in childhood were at higher suicide risk (*P* < 0.01). Multiple linear regression analysis also indicated that being female (β = 1.229, *P* < 0.001) was a factor in suicide risk. In addition, the severity of teenagers' depression (β = 0.205, *P* < 0.001) was associated with higher suicide risk, while better family function (β = −0.252, *P* < 0.001) and higher subjective wellbeing (β = −0.361, *P* < 0.001) were associated with lower suicide risk (Table 2).

**Table 1:** Difference in the suicide risk by demographic characteristics among teenagers with a history of self-harm behaviors.

**Variables**	**The score of SBQ-R (mean ±SD)**	***F*/*t***	***P***
**Grade**		1.176	0.240
Junior high school (*n* = 394)	9.82 ± 3.78		
Senior high school (*n* = 519)	9.53 ± 3.62		
**Gender**		−6.472	**<0.001**
Male (*n* = 316)	8.59 ± 3.75		
Female (*n* = 597)	10.22 ± 3.54		
**The status of only child**		0.599	0.549
No (*n* = 340)	9.75 ± 3.75		
Yes (*n* = 573)	9.6 ± 3.66		
**Childhood with parents**		3.121	**0.002**
No (*n* = 315)	10.18 ± 3.76		
Yes (*n* =5 98)	9.38 ± 3.63		
**The education degree of father**		0.249	0.779
High school and below (*n* = 349)	9.59 ± 3.62		
College and undergraduate (*n* = 485)	9.73 ± 3.76		
Master's degree or above (*n* = 79)	9.47 ± 3.59		
**The education degree of mother**		0.833	0.436
High school and below (*n* = 373)	9.47 ± 3.58		
College and undergraduate (*n* = 485)	9.78 ± 3.74		
Master's degree or above (*n* = 55)	9.85 ± 4.03		
**Annual household income**		1.927	0.124
<80,000 (*n* = 152)	9.34 ± 3.67		
80,000–150,000 (*n* = 323)	9.78 ± 3.63		
150,000–300,000 (*n* = 274)	9.39 ± 3.63		
>300,000 (*n* = 164)	10.14 ± 3.90		

The bold values indicates the emphasis on *P* values < 0.001.

**Table 2:** Factors associated with suicide risk using multiple linear regression analysis.

**Variables**	**β**	**S.E**.	***t***	***P***
Grade (senior vs. junior high school)	0.275	0.211	1.306	0.192
Gender (female vs. male)	1.229	0.219	5.617	**<0.001**
The status of only child (yes vs. no)	0.258	0.221	1.169	0.243
Childhood with parents (yes vs. no)	−0.123	0.219	−0.562	0.574
The education degree of father	−0.318	0.225	−1.415	0.157
The education degree of mother	0.175	0.237	0.741	0.459
Annual household income	0.135	0.124	1.093	0.275
Family function	−0.252	0.04	−6.225	**<0.001**
Depression of parents	0.005	0.037	0.144	0.886
Anxiety of parents	−0.019	0.04	−0.486	0.627
Subjective wellbeing	−0.361	0.087	−4.129	**<0.001**
Depression of student	0.205	0.029	7.177	**<0.001**
Anxiety of student	0.047	0.032	1.448	0.148

The bold values indicates the emphasis on *P* values <0.001.

### 3.2. Correlation between factors associated with suicide risk

The full pattern of correlations is summarized in Table 3. The results showed that family function and subjective wellbeing were negatively correlated with suicide risk (*P* < 0.001), and the severity of teenagers' depression was positively correlated with suicide risk (*P* < 0.001). Moreover, a significant positive correlation was found between family function and subjective wellbeing (*P* < 0.001). Family function and subjective wellbeing showed significant negative correlations with the severity of teenagers' depression as well (*P* < 0.001).

**Table 3:** The association between family and emotional factors with the risk of suicide.

**Variables**	**Suicide risk**	**Family function**	**Subjective wellbeing**
Family function	−0.412^**^	1	–
Subjective wellbeing	−0.422^**^	0.386^**^	1
Depression of student	0.561^**^	−0.389^**^	−0.505^**^

^**^P < 0.001.

### 3.3. The chain mediation of subjective wellbeing and depression in the relationship between family function and suicide risk

**Figure 1:**
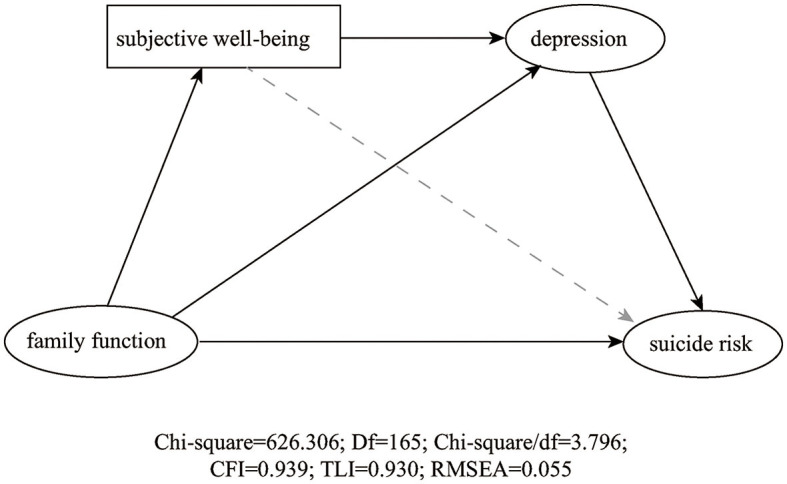
Structural equation model. The solid line represents a significant relationship between the two variables. Chi-square = 626.306; Df = 165; Chi-square/df = 3.796; CFI = 0.939; TLI = 0.930; RMSEA = 0.055.

As shown in Table 4 and Figure 1, when the variable of gender was controlled, family function was negatively associated with suicide risk (95%CI: −0.324, −0.173) and depression was positively associated with suicide risk (95%CI: 0.415, 0.584), while subjective wellbeing was not significantly associated with suicide risk (*P* = 0.069, 95%CI: −0.149, 0.005). In addition, the results of the chain mediation model showed that depression acted as an important mediator, as the chain mediating effect of depression and the sequential chain mediating effect for subjective wellbeing and depression in the association between family function and suicide risk were both significant (95%CI: −0.171, −0.084; 95%CI: −0.119, −0.070). The results of the SEM showed that the chi-square test of model fit value was 626.306, degrees of freedom = 165, RMSEA = 0.055, CFI = 0.939, and TLI = 0.930, indicating a good fit.

**Table 4:** Relationships among multiple variables in the structural equation model.

**Relationship**	**Estimate**	**S.E**.	***P***	**95%CI**
F → SWB	0.413	0.033	**<0.001**	0.348, 0.478
SWB → D	−0.449	0.035	**<0.001**	−0.517, −0.382
F → D	−0.249	0.037	**<0.001**	−0.321, −0.176
F → S	−0.246	0.038	**<0.001**	−0.324, −0.173
SWB → S	−0.074	0.04	0.063	−0.149, 0.005
D → S	0.503	0.042	**<0.001**	0.415, 0.584
F → SWB → S	−0.030	0.017	0.069	−0.064, 0.002
F → D → S	−0.125	0.022	**<0.001**	−0.171, −0.084
F → SWB → D → S	−0.093	0.012	**<0.001**	−0.119, −0.070

F, family function; D, depression; SWB, subjective wellbeing; S, suicide; S.E., standard error; CI, confidence interval. The bold values indicates the emphasis on *P* values <0.001.

## 4. Discussion

Individuals with previous self-harm behaviors have been regarded as a high-risk population for suicide (39). Our study showed that up to 78.6% of teenagers with a history of self-harm behaviors might have the possibility of suicide, the rate was much higher than that of general teenagers (40). This study also found that gender, family function, subjective wellbeing, and severity of teenagers' depression were associated with the suicide risk of teenagers with a history of self-harm behaviors. Additionally, the results of the SEM showed that family function, subjective wellbeing, and severity of depression affected the suicide risk of teenagers with a history of self-harm behaviors via chain mediation.

Consistent with previous studies, gender differences played important roles in adolescents' suicide (41). A potential reason might be that compared to males, females are more likely to be frustrated, guilty, and less confident during the adolescence transition period, and they tend to react to depression in a ruminative manner (42), consequently, they are more prone to extreme thoughts and behaviors. Another explanation might be that males tend to choose more violent and lethal methods, such as firearms and hanging, rendering males presenting with fewer suicide attempts but higher suicide death (43).

In this study, family function was found to be negatively associated with suicide risk in teenagers with a history of self-harm behaviors, both independently and via chain mediation of subjective wellbeing and depression, highlighting the role of family factors in the mitigation of suicide risk. The relationship between family function and suicide risk has been highlighted, as family characteristics including high family conflict and low parental monitoring are related to a higher risk of death by suicide (19, 44). Alvarez et al. pointed out that dysfunctional family patterns, such as parental neglect, affection-less control bonding, and insecure attachment were significant risk factors for suicide, conversely, parental care and sense of security were revealed as protective factors for suicide (45). Good family function, such as family cohesion, was related to less suicide risk, even though this association was conditioned by self-stigma (46). Susukida et al. also found that regardless of having lived with parents during childhood, individuals who perceived parental love had significantly lower lifetime suicide risk (47). Furthermore, family therapy has been considered a recommended approach for decreasing suicide, especially among children and adolescents (48, 49). For example, attachment-based family therapy and family-enhanced non-directive supportive therapy substantially reduced adolescents' suicide ideation and depressive symptoms, better than other more intensive, multicomponent treatments (48). Therefore, improving family function seems to be an essential target for suicide prevention among teenagers.

Previous self-harm behaviors could be an earlier signal of depression, and, in turn, depression might lead to future suicide in the face of family pathology (50). Affective states might confer risk for suicide, particularly, patients with severe depression symptoms presented more frequent and less controlled suicide ideation (51, 52). Furthermore, depressive symptom severity might serve as a mediator between family function and suicide ideation severity (53). Our results suggested that there might be chain mediation effects, namely, depression mediates the suicide risk between family function and subjective wellbeing, and to our knowledge, this has been rarely reported. Subjective wellbeing is a psychological index to comprehensively measure an individual's satisfaction with their life and the prevalence of positive emotions over negative emotions (54). Previous studies have suggested that subjective wellbeing was negatively associated with suicide risk (30, 31). Whereas, a direct association between subjective wellbeing and suicide risk was not observed in this study, which might be masked by depression symptoms. Family has been examined as a typical microsystem that influences an individual's subjective wellbeing, and ~40% of the variation in children's subjective wellbeing could be explained by family factors (55–57). The underlying mechanisms of the chain mediation effects might be that impaired family function creates a stressful environment, increasing the feelings of loneliness and abandonment, which then decrease children and adolescents' subjective wellbeing perception, further aggravating their emotion regulation difficulties, and finally, leading to suicide (58–60).

Overall, the current study suggested that family function, severity of depression, and subjective wellbeing were important factors that affected suicide risk in teenagers with a history of self-harm behavior. More importantly, depression mediated the suicide risk between family function and subjective wellbeing. Hence, comprehensive intervention strategies consisting of multilevel approaches, such as programs enhancing family function, depression education, and subjective wellbeing promotion, should be recommended in the prevention of suicide risk.

Several limitations of this study need to be considered. First, this study was cross-sectional and unable to show causality. Second, this study focused on the history of self-harm behaviors without investigating their frequency, more detailed clues might be lost. Third, the SBQ-R has been mainly applied to Chinese college students with good reliability and validity, while its reliability and validity among Chinese teenagers should be further evaluated. Finally, study participation was voluntary and non-mandatory and depended on the cooperation and willingness of participants; therefore, a certain degree of non-response bias might exist in the present results.

## 5. Conclusion

Among teenagers with a history of self-harm behaviors, 78.6% were regarded to have the possibility of suicide. Family function, depression, and subjective wellbeing were associated with suicide risk, and these associated factors affect the suicide risk via chain mediation. Consequently, intervention strategies such as depression education as well as programs promoting family function and subjective wellbeing should be applied to prevent the suicide of teenagers with a history of self-harm behaviors.

## Data availability statement

The raw data supporting the conclusions of this article will be made available by the authors, without undue reservation.

## Ethics statement

The studies involving human participants were reviewed and approved by the Ethics Committee of the Wuhan Mental Health Center. Written informed consent to participate in this study was provided by the participants' legal guardian/next of kin.

## Author contributions

MC was responsible for the study design, statistical analyses, and manuscript writing and submission. YZ was responsible for the draft of the protocol and recruitment of participants. DL was responsible for the writing—review and funding acquisition. SY, ML, MW, and XL were responsible for the evaluation of mood symptoms and data collection. BY was responsible for methodology and funding acquisition. YL was responsible for project administration and resources. LL was responsible for project supervision, conceptualization, writing—review, and editing. All authors contributed to the article and approved the submitted version.
